# Childhood multidrug-resistant tuberculosis in the European Union and European Economic Area: an analysis of tuberculosis surveillance data from 2007 to 2015

**DOI:** 10.2807/1560-7917.ES.2017.22.47.17-00103

**Published:** 2017-11-23

**Authors:** Csaba Ködmön, Martin van den Boom, Phillip Zucs, Marieke Johanna van der Werf

**Affiliations:** 1 European Centre for Disease Prevention and Control, Stockholm, Sweden; 2 Joint Tuberculosis, HIV and Viral Hepatitis Programme, World Health Organization, Regional Office for Europe, Copenhagen, Denmark

**Keywords:** multidrug resistance, tuberculosis, paediatric tuberculosis, extrapulmonary tuberculosis, European Union

## Abstract

Confirming tuberculosis (TB) in children and obtaining information on drug susceptibility is essential to ensure adequate treatment. We assessed whether there are gaps in diagnosis and treatment of multidrug-resistant (MDR) TB in children in the European Union and European Economic Area (EU/EEA), quantified the burden of MDR TB in children and characterised cases. **Methods**: We analysed surveillance data from 2007 to 2015 for paediatric cases younger than 15 years. **Results**: In that period, 26 EU/EEA countries reported 18,826 paediatric TB cases of whom 4,129 (21.9%) were laboratory-confirmed. Drug susceptibility testing results were available for 3,378 (17.9%), representing 81.8% of the confirmed cases. The majority (n = 2,967; 87.8%) had drug-sensitive TB, 249 (7.4%) mono-resistant TB, 64 (1.9%) poly-resistant TB, 90 (2.7%) MDR TB and eight (0.2%) had extensively drug-resistant (XDR) TB. MDR TB was more frequently reported among paediatric cases with foreign background (adjusted odds ratio (aOR) = 1.73; 95% confidence interval (95% CI): 1.12–2.67) or previous TB treatment (aOR: 6.42; 95% CI: 3.24–12.75). Successful treatment outcome was reported for 58 of 74 paediatric MDR TB cases with outcome reported from 2007 to 2013; only the group of 5–9 years-olds was significantly associated with unsuccessful treatment outcome (crude odds ratio (cOR) = 11.45; 95% CI: 1.24–106.04). **Conclusions**: The burden of MDR TB in children in the EU/EEA appears low, but may be underestimated owing to challenges in laboratory confirmation. Diagnostic improvements are needed for early detection and adequate treatment of MDR TB. Children previously treated for TB or of foreign origin may warrant higher attention.

## Introduction

Multidrug-resistant tuberculosis (MDR TB) is a major challenge in the fight to end the global tuberculosis (TB) epidemic [1]. Of 10.4 million incident cases of TB and 1.8 million deaths from TB estimated for 2015 globally, ca 480,000 cases and ca 190,000 deaths were attributed to MDR TB [2]. An estimated 1.0 million incident cases of TB and 170,000 deaths from TB occurred in children [2]. The estimated proportion of MDR TB among children with TB in 2014 was 2.9% (range: 2.7–3.1) [3]. In the European Union (EU) and European Economic Area (EEA), children under 15 years of age accounted for 2,415 (4.2%) of 57,136 TB cases notified in 2015, which corresponded to a notification rate of 3.0 per 100,000 population [4]. Data on drug resistance patterns in children in the EU/EEA have, to our knowledge, not been analysed in the past.

Despite the availability of modern technologies such as nucleic acid amplification tests, diagnosis of TB in children is challenging. Clinical diagnosis is not standardised [5,6], and the traditional and molecular diagnostic methods lack sensitivity in children, while serological methods lack specificity [6]. Also, since most laboratory tests for TB are done on sputum and children most often have paucibacillary disease, it is not easy to identify *Mycobacterium tuberculosis* bacilli or DNA in their sputum samples. Furthermore, specimens for culture and drug susceptibility testing are often difficult to obtain, particularly from the youngest (younger than 3 years) who cannot expectorate sputum [7,8]. In such cases, hospital clinicians may obtain specimens through gastric aspirates, induced sputum and/or nasopharyngeal aspirates, and bronchoalveolar lavage [6]. Moreover, in children, *M. tuberculosis* tends to spread from the lungs to the regional hilar and mediastinal lymph nodes as well as to other parts of the body; therefore, extrapulmonary TB, which is more difficult to diagnose than pulmonary TB, is more common than in adults [7]. Despite rigorous specimen collection and laboratory techniques, culture confirmation of pulmonary TB succeeds in no more than 40% of children [6] and even less frequently for extrapulmonary TB [8,9]. Because conventional drug susceptibility testing (DST) is performed by phenotypic methods that depend on culture confirmation, DST results are often lacking in children [6,10].

First-line drugs for treating active TB disease in adults and children are isoniazid, rifampicin, pyrazinamide and ethambutol [11-13]. These drugs are generally better tolerated by children than adults [14]. Since DST results are often not available for children, they risk receiving suboptimal treatment and facing an unfavourable treatment outcome [15].

The published literature lacks robust epidemiological information on diagnosis and treatment of MDR TB in children, which may be due to challenges in diagnosis and under-detection [16]. The little evidence available largely consists of case reports or case series reports, mainly from countries outside Europe [17-22]. Therefore, we aimed to describe the burden of and identify factors associated with MDR TB in children and to assess whether there are gaps in diagnosis and treatment of MDR TB in children.

## Methods

The European Centre for Disease Prevention and Control (ECDC) has been collecting case-based TB surveillance data from EU and EEA countries since 2007 and storing them in a common database, the European Surveillance System (TESSy). Designated national surveillance institutions are responsible for data reporting to ECDC and data validation. The detailed data collection methods, definitions and figures on data completeness are described elsewhere [4].

TB cases were defined according to the case definition published by the European Commission [23], and all confirmed, probable and possible cases were included in the analysis. Liechtenstein only reported case-based TB surveillance data for 2007 and Croatia for 2012, 2013, 2014 and 2015; both countries were therefore excluded from the analysis. France, Italy and Spain are not reporting case-based DST data to ECDC and were also excluded. Surveillance data reported by the remaining 26 EU/EEA countries and covering the period from 2007 to 2015 were extracted from the TESSy database on 10 October 2016. Greece did not report treatment outcome after 12 months for the years 2007 to 2012 and was therefore excluded from the treatment outcome analysis.

TB cases younger than 15 years were considered paediatric TB cases. MDR TB was defined as resistance to at least isoniazid and rifampicin and extensively drug-resistant (XDR) TB as resistance to at least isoniazid and rifampicin, any fluoroquinolone and any of the three second-line injectable drugs amikacin, capreomycin and kanamycin. DST was considered completed if a TB case was laboratory-confirmed according to the EU case definition (culture-positive or microscopy-positive for acid-fast bacilli and nucleic acid-positive for TB) [23] and resistance data for at least isoniazid and rifampicin were available. Cases with both pulmonary and extrapulmonary TB were classified as pulmonary TB. For the majority of countries, geographic origin was considered foreign if place of birth was outside the reporting country. For Austria, Belgium, Greece, Poland and Hungary from 2010 onwards and for Malta in 2010, foreign origin was defined as a citizenship other than that of the reporting country. For cases with TB sensitive to isoniazid or rifampicin, treatment outcome data (success, failed, died, lost to follow-up, still on treatment) were collected 12 months after the start of treatment. For MDR and for XDR TB cases, treatment outcome was collected 24 months and 36 months after start of treatment, respectively. Treatment outcome was considered successful if a case was cured or had completed treatment.

We analysed paediatric TB cases by laboratory confirmation and drug resistance (susceptible, monoresistant, polyresistant, MDR (excluding XDR) and XDR), age group (0–4, 5–9 and 10–14 years), sex, origin (native, foreign), TB site (pulmonary, extrapulmonary), treatment history (new or previously treated case) and treatment outcome. Susceptible, monoresistant and polyresistant TB cases were considered as non-MDR TB cases. 

Factors associated with laboratory confirmation, multidrug resistance and treatment outcome were identified by univariate analysis. If for any univariate association, the p value was less than or equal to 0.1 by chi-squared test, the variable was included in a multivariable logistic regression model applying backward elimination based on maximum likelihood estimates. Statistical associations were expressed as odds ratios (OR) with 95% confidence intervals (CI). Differences were considered statistically significant if p < 0.05 as determined by chi-squared test. Statistical analysis was performed using STATA 14 software (StataCorp, Texas, United States (US)).

## Results

From 2007 to 2015, 18,826 paediatric TB cases were reported in the 26 EU/EEA countries included in this study. The average percentage of paediatric TB cases among all notified TB cases was 3.7% over the years (range: 3.5–3.8). 

Among the 18,826 notified paediatric TB cases, 4,129 (21.9%) were laboratory-confirmed, of whom 894 (21.6%) had a positive NAAT test and DST results were available for 3,378 (17.9%), representing 81.8% of all laboratory-confirmed paediatric TB cases. The majority of cases for whom DST had been done (n = 2,967; 87.8%) had drug-sensitive TB, 249 (7.4%) had monoresistant TB, 64 (1.9%) polyresistant TB, 90 (2.7%) MDR TB (excluding XDR TB), and eight (0.2%) were diagnosed with XDR TB.

**Figure 1:**
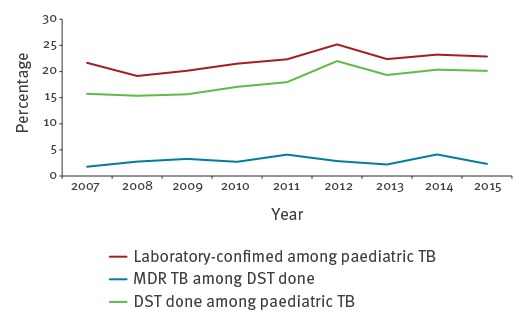
Drug susceptibility testing coverage and percentage of multidrug-resistant tuberculosis among children in 26 European Union and European Economic Area countries reporting case-based drug susceptibility data, 2007–2015 (n = 18,826)

DST: drug susceptibility testing; MDR: multidrug-resistant; TB: tuberculosis.

The percentage of MDR TB among paediatric TB cases with available DST results ranged between 1.8% and 4.1% during the study period, while the DST coverage among all paediatric TB cases was between 15.4% and 22.0% in the different years (Figure 1). The trend of paediatric MDR TB among cases with available DST results and the trend of DST coverage of paediatric TB cases was stable over the study period.

**Figure 2:**
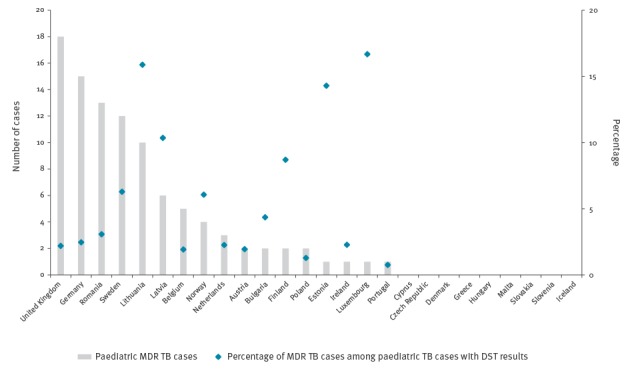
Number of and percentage paediatric multidrug-resistant tuberculosis cases among laboratory-confirmed paediatric TB cases with drug susceptibility testing results, European Union and European Economic Area, 2007–2015 (n = 3,378)

DST: drug susceptibility testing; MDR: multidrug-resistant; TB: tuberculosis.

The highest number of paediatric MDR TB (including XDR TB) cases was reported by the United Kingdom (UK), followed by Germany, Romania and Sweden; nine countries did not report any paediatric MDR TB case during the study period (Figure 2). Countries with the highest number of paediatric MDR TB cases were countries with a large population such as Germany or the UK, countries with comparatively higher burden of TB such as Romania, or countries with a proportionately high migrant population such as Sweden. Country-specific percentages of paediatric MDR TB among all paediatric TB cases ranged from 0.8% in Portugal to 16.7% in Luxembourg.

MDR TB was associated with foreign origin (aOR = 1.73; 95% CI: 1.12–2.67) and previous TB treatment (aOR = 6.42; 95% CI: 3.24–12.75) (Table).

**Table:** Factors associated with paediatric multidrug-resistant tuberculosis in 26 European Union and European Economic Area countries reporting case-based drug-susceptibility data, 2007–2015 (n = 3,378)

	Paediatric MDR TB cases		Paediatric non-MDR TB cases		Univariate logistic regression	Multivariable logistic regression
	n	%	n	%	OR (95% CI)	OR (95% CI)
Total	98	2.9	3,280	97.1	Not done	Not done
*Age groups (n = 3,378) *						
0–4 years	26	26.5	1,156	35.2	1	Not included
5–9 years	22	22.5	577	17.6	1.69 (0.95–3.02)	Not included
10–14 years	50	51.0	1,547	47.2	1.43 (0.89–2.32)	Not included
*Sex (n = 3,372) *						
Male	41	41.8	1,567	47.9	1	Not included
Female	57	58.2	1,707	52.1	1.28 (0.85–1.92)	Not included
*Origin (n = 3,315) *						
Native	53	54.1	2,163	67.2	1	1
Foreign	45	45.9	1,054	32.8	1.74 (1.16–2.61)	1.73 (1.12–2.67)
*TB treatment history (n = 2,949) *						
New	76	87.4	2,798	97.8	1	1
Previously treated	11	12.6	64	2.2	6.33 (3.21–12.48)	6.42 (3.24–12.75)
*Site of disease (n = 3,374) *						
Pulmonary	68	69.4	2,448	74.7	1	Not included
Extrapulmonary	30	30.6	828	25.3	1.30 (0.84–2.02)	Not included

CI: confidence interval; MDR: multidrug-resistant; OR: odds ratio; TB: tuberculosis.

**Figure 3:**
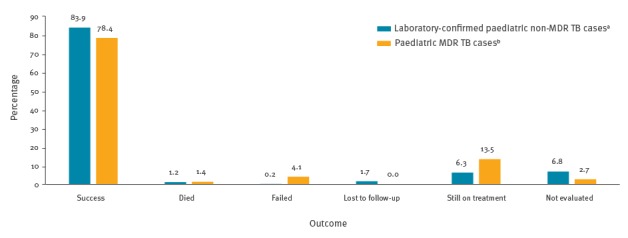
Treatment outcome in paediatric tuberculosis cases by MDR status, European Union and European Economic Area, 2007–2014 (n = 2,980)

MDR: multidrug-resistant; TB: tuberculosis.

^a^ Treatment outcome after 12 months, cohort 2007–14.

^b^ Treatment outcome after 24 months, cohort 2007–13.

Successful treatment outcome after 24 months of treatment was reported for 58 of 74 paediatric MDR TB cases notified from 2007 to 2013 and successful treatment outcome after 12 months of treatment was reported for 2,438 (83.9%) of 2,906 laboratory-confirmed paediatric non-MDR TB notified from 2007 to 2014 (Figure 3).

Non-MDR TB: susceptible, monoresistant and polyresistant TB; MDR TB: MDR TB including XDR TB.

For paediatric MDR TB, treatment success was less frequently reported in children aged 5–9 years than in those younger than 5 years (OR = 11.45; 95% CI: 1.24–106.04).

## Discussion

We used surveillance data to describe the burden of MDR TB in children in 26 EU/EEA countries from 2007 to 2015 and to assess whether there are gaps in diagnosis and treatment of MDR TB in children. Our analysis showed that 2.9% of all paediatric TB cases with DST results were diagnosed with MDR or XDR TB. This is similar to the proportions reported from England and Wales (2.3%) and the US (2.3%) in children under 5 years of age [24,25]. Proportions of MDR TB were slightly higher in all notified TB cases (i.e. TB cases regardless of the age) in the EU/EEA, i.e. 4.1% [4]. Only 21.9% of the reported paediatric TB cases in EU/EEA were laboratory-confirmed and could thus undergo DST. Of all notified TB cases in the EU/EEA, ca 80% were laboratory-confirmed in 2015 [4]. In other upper middle- and high-income countries, e.g. England and Wales [24], the US [25,26] and Australia [27], the proportion of paediatric TB cases that is laboratory-confirmed is comparable. Thus, the challenges in other countries and World Health Organization (WHO) regions seem to be similar. In our analysis, almost half of the paediatric TB cases had extrapulmonary TB, a proportion that has also been described for other settings [9,28-32].

The high percentage of paediatric TB cases without laboratory confirmation raises the question of what the undiagnosed fraction of MDR TB is in children. The need for improvements in laboratory diagnosis of paediatric TB has been acknowledged and features prominently in the WHO roadmap for childhood tuberculosis [33]. A mathematical modelling study suggested far more MDR TB among children than diagnosed globally, with the burden of paediatric MDR TB being correlated with the overall MDR TB burden in a particular region [3].

DST results were reported for most paediatric TB cases who were laboratory-confirmed in the analysed EU/EEA countries. Accurate and comprehensive DST information is the cornerstone for prescribing adequate TB treatment. If such DST information is not available, treatment can only be based on the drug resistance pattern of the probable source case which, in low-incidence settings, is believed to be reflected in the resistance pattern of the secondary case [29]. In higher-incidence countries, more than half of the drug susceptibility patterns of household contacts may match the pattern of the purported source [34]. Moreover, the source case may not always be known. In children younger than 5 years in the US, only 53% had a known source case and could thus be treated adequately without information on drug susceptibility [25].

In line with other studies, we found foreign origin and previous TB treatment history to be significantly associated with paediatric MDR TB [30,35]. A history of previous TB treatment suggests potentially preventable acquired drug resistance or inadequate treatment of primary drug-resistant TB, while the frequency of MDR TB among children with foreign origin reflects the prevalence of MDR TB in the respective country of origin.

In the EU/EEA, most paediatric MDR/XDR TB cases notified in 2007-2013 were successfully treated, with one death and two failed treatments reported. This indicates that in EU/EEA countries, the main challenge lies in the identification of paediatric MDR/XDR TB cases and that once cases have been detected, means and expertise are sufficient to ensure adequate treatment. The treatment success rate was lower than in South Africa (82–90%) [35,36] and Israel (97.8%) [37]. In our study, half of the successful treatment outcomes in paediatric MDR TB cases were in the age group of 10–14 year-olds, which can be attributed to increasing tolerance of the TB treatment with age [38]. Only in the age group of 5–9 year-olds, a significantly lower percentage of paediatric MDR TB cases with treatment success was identified, although the analysis was limited by the small number of cases.

## Limitations

An important limitation of the study is that information on TB contact history and preventive treatment of TB contacts is not collected at EU/EEA level. Therefore, epidemiological links cannot be identified and the effectiveness of the preventive TB treatment cannot be assessed. The incompleteness of reported TB notification data, the differences in accuracy of laboratory diagnosis of paediatric TB between EU/EEA countries and the heterogeneity of the TB situation in the EU/EEA should be taken into account when interpreting the results.

## Conclusions

The burden of MDR TB in children in the EU/EEA appears low and stable over time. Laboratory confirmation of paediatric TB remains a challenge in EU/EEA and may lead to an underestimation of the real burden of MDR TB among children. Improvements in the laboratory diagnosis of paediatric TB are needed for early detection and adequate treatment of MDR TB. Children previously treated for TB and children of foreign origin may need to be given higher attention regarding MDR TB.
